# Revising Computerized Therapy for Wider Appeal Among Adolescents: Youth Perspectives on a Revised Version of SPARX

**DOI:** 10.3389/fpsyt.2019.00802

**Published:** 2019-11-22

**Authors:** Theresa M. Fleming, Karolina Stasiak, Emma Moselen, Eve Hermansson-Webb, Matthew Shepherd, Mathijs Lucassen, Lynda M. Bavin, Sally Nicola Merry

**Affiliations:** ^1^Department of Psychological Medicine, The University of Auckland, Auckland, New Zealand; ^2^School of Health, Victoria University of Wellington, Wellington, New Zealand; ^3^School of Psychology, Massey University, Auckland, New Zealand; ^4^School of Health, Wellbeing and Social Care, The Open University, Milton Keynes, United Kingdom

**Keywords:** digital therapy, computerized cognitive behavior therapy, adolescent, depression, prevention, internet interventions

## Abstract

**Background:** The way in which computerized therapy is presented may be important for its uptake. We aimed to explore adolescents’ views on the appeal of a tested computerized cognitive behavioral therapy (CCBT) for depression (SPARX), and a revised version (SPARX-R). The versions were similar but while SPARX is presented explicitly as a treatment for depression, SPARX-R is presented as providing skills that could be useful for young people for when they were depressed, down, angry, or stressed.

**Methods:** We held 9 focus groups with a total of 79 adolescents (13–19 years old; 47 females; 34 New Zealand European; 22 Māori or Pacific; 60 reported having experienced feeling down or low for at least several days in a row). Groups viewed the opening sequences of SPARX and SPARX-R (in random order), then took part in a semi-structured discussion and completed a brief questionnaire. Responses were analyzed using a general inductive approach.

**Results:** Participants considered both SPARX and SPARX-R useful and considered the stated purpose of the program to be important. Four themes contrasted the two approaches. The first, “naming depression is risky”, referred to perceptions that an explicit focus on depression could be off-putting, including for adolescents with depression. The second theme of “universality” reflected preferences for a universal approach as young people might not recognize that they were depressed, and all would benefit from the program. In contrast, “validation” reflected the view of a significant minority that naming depression could be validating for some. Finally, the theme of “choice” reflected a near-unanimously expressed preference for both options to be offered, allowing user choice. In questionnaire responses, 40 (68%) of participants preferred SPARX-R, 13 (18%) preferred SPARX, while 10 (14%) “didn’t mind”. Responses were similar among participants who reported that they had experienced at least a few days of low mood and those who had not.

**Conclusions:** The way a CCBT program is presented may have implications for its appeal. The potential population impact of CCBT programs explicitly targeting depression and those targeting more universal feelings such as being stressed or feeling depressed should be explored for varied user groups.

## Introduction

Depression and sub-threshold depression are common and disabling, with up to 25% of young people experiencing depression by the end of adolescence (1). Cognitive behavioral therapy (CBT) is a first-line treatment (2), but the majority of adolescents do not use professional services despite significant symptoms (3, 4). Notably, many young people, particularly those from minority groups or cultures, report that they would prefer to access support from familiar people, use self-help or internet-based information, or that they would not seek any help (5–7).

Computerized cognitive behavior therapy (CCBT) has been demonstrated to be effective in alleviating depression in adolescents (8–10). Online approaches have the potential to reduce obstacles to therapy associated with location, cost, and convenience, and may reduce obstacles associated with stigma and limited help-seeking skills (11, 12). Our youth e-therapy team (SM, KS, TF, ML, MS) developed and tested SPARX (smart, positive, active, realistic, X-factor thoughts), an interactive CCBT program for adolescents with mild-to-moderate depression (13). SPARX comprises seven levels, which teach CBT skills such as relaxation, problem solving, and recognizing and challenging negative automatic thoughts *via* direct instruction and play-based learning in a fantasy environment. In a randomized controlled trial with 187 adolescents with symptoms of depression, SPARX was found to be non-inferior to treatment-as-usual (13). In smaller trials, it was found to be more effective than waitlist control for students excluded from mainstream education (14), appealing to indigenous Māori young people (15) and, in a “rainbow” version, promising for sexual minority youth (16). SPARX met with high approval from adolescents and providers (13, 17, 18).

However, analysis of data from our research highlighted some potential problems with targeting CCBT explicitly toward “youth with depression”. First, some front-line workers who support young people were not confident about identifying depression and did not wish to do so (18). Consistent with social service providers (19), these helping professionals saw a diagnostic and treatment approach as inconsistent with their role, which they considered to be supportive, normalizing, and non-pathologizing. Many considered that identifying adolescents on the basis of their mental health problems might be unwanted and unhelpful. Second, adolescents themselves reported that they might not recognize that they were depressed or down. In addition, even if they did recognize this, they would not ask for help and would resist receiving help that differentiated them from their peers (12, 17). Third, in a pragmatic trial of SPARX CCBT in alternative education settings, a universal approach appeared advantageous. In this study, the recruitment and retention of participants (both with and without symptoms) was much higher in the five study sites that invited all students to participate than in the single site that used a targeted approach (14). Participating adolescents also reported that they found CCBT helpful, whether or not they had depressive symptoms at baseline (17).

There is promising evidence in support of CBT-based prevention programs, particularly with targeted or indicated groups. Meta-analyses show that psychological depression prevention programs for adolescents, many based on CBT principles, are promising for preventing depression compared with no intervention, with a number of studies showing a decrease in episodes of depressive illness during the year following intervention (20, 21). In addition, there is evidence that CCBT interventions can be effective in improving sub-threshold symptoms (22) and preventing mental disorders among adults (23).

Based on our findings and this evidence, we developed a revised version of SPARX, directed at preventing and treating sub-clinical symptoms as well as treating mild-to-moderate depression. TF, an experienced youth mental health clinician and SPARX co-developer, instigated the development of SPARX-R with the support of other SPARX co-developers SM, KS, ML, and MS (SM is a consultant child and adolescent psychiatrist and led the development of SPARX, ML and MS are experienced child and adolescent therapists and researchers, and KS is a research psychologist). All developers reviewed SPARX content and proposed changes and TF consulted adolescent advisors and an external clinical team on the proposed alterations and wording. SPARX developers reached consensus on the revised script in an iterative fashion. Key SPARX content is highlighted in **Table 1**. Almost all of this content was identified as appropriate or acceptable for SPARX-R, with the following alterations:

**Table 1:** Revising SPARX.

Key SPARX content	Appropriate for SPARX-R	Acceptable for SPARX-R	Amended for SPARX-R
Welcome and virtual rapport building			Yes*
Psychoeducation regarding: depression being a common challenge that is amenable to change; methods of dealing with depression; linking thoughts, actions, and feelings Introducing a “shield against depression”			Yes** Yes***
Expression of hope		Yes	
Cognitive restructuring: recognizing and challenging negative automatic thoughts, identifying and promoting SPARX (smart, positive, active, realistic, X-factor thoughts)		Yes	
Relaxation skills: controlled breathing, progressive muscle relaxation	Yes		
Activity scheduling	Yes		
Interpersonal skills: social skills, listening skills, assertion and negotiation skills	Yes		
Dealing with strong emotions: anger and hurt feelings, distress tolerance	Yes		
Problem solving	Yes		
Keeping on trying, asking for help, overcoming barriers to change	Yes		

*Welcome amended as described in text.

**Psychoeducation amended to feeling down, stressed, or depressed being a common challenge that is amenable to change; methods of dealing with these feelings when they become overwhelming or go on for a long time; linking thoughts, actions, and feelings.

***Amended to a “shield against feeling down”.

- SPARX begins with a “guide” character (a virtual therapist), who states that the purpose of the program is to help young people “who feel down or depressed”. The term “depression” is thereafter used frequently, with the assumption that the user has depression.
- In SPARX-R, the guide states: “This version of SPARX was made to help young people who are having hassles and feeling down, stressed, or angry a lot of the time. Even if you are doing fine, SPARX can help strengthen your skills for dealing with problems when they do come along.”
- The term “depression” was replaced in SPARX-R by broader terminology, most often “feeling down, stressed, or angry a lot of the time”.

To date SPARX-R has been tested in two small pilots and one cluster randomized controlled trial. The first prototype (SPARX-R 1.0) was piloted in two small studies using compact disc read-only memory (CD-ROMs) or memory sticks. The first pilot, in a New Zealand youth justice program, was unsuccessful. Few participants attended the program regularly, and even fewer began SPARX-R, with no effects reported (24). The second was held in Irish “Youthreach” alternative education centers. There were many technical problems with SPARX-R (e.g., downloads, saving progress). Despite these issues, there was an effect for emotion regulation, but no effect for depression was reported (25). In the next prototype (SPARX-R 1.1) the main technical issues were addressed and SPARX-R was delivered online in a cluster randomized controlled trial of 540 final year students in 10 Australian high schools. Participants in the SPARX-R arm of the study (n = 242) showed significantly reduced depressive symptoms relative to control participants (n = 298) at post-intervention (26). In contrast, a school based prevention trial in the Netherlands, found that a Dutch translation of SPARX was no more effective for reducing subclinical depressive symptoms among girls than a weekly detailed monitoring control condition or group-based CBT (27).

Despite these developments, adolescents’ views on the relative relevance and appeal of SPARX and SPARX-R have not been explored. In this study we aimed to address this gap. In particular, we sought to understand whether framing the program as explicitly “for depression” or for youth more generally was salient to participants and implications for implementation of digital mental health tools for youth.

## Methods

As user preferences regarding the framing and focus of online therapy are relatively unexplored, we carried out a qualitative study using focus groups to explore adolescents’ views. Focus groups are ideal for exploring people’s experiences of health services and allow the researchers to investigate both convergent and individual views (28). To ensure that all participants’ opinions were sufficiently captured, including points that they may not wish to raise in front of peers, participants completed a brief pen-and-paper questionnaire at the end of the focus group. Ethics approval was obtained from the University of Auckland Human Participants Ethics Committee (Reference 2015/014991).

### Procedure

We approached 10 schools and 3 community organizations in ethnically and socio-economically diverse areas of Auckland, New Zealand. School principals and organization managers gave assent for adolescents to be invited to participate and were given material to disseminate to adolescents and their parents/caregivers through their standard communication channels. Interested students were given information and encouraged to take this home. Parents/caregivers of adolescents younger than 16 could opt to have their children excluded. On the day of the focus groups adolescents gave their own written consent to participate. Participants were offered a cinema voucher as an acknowledgement of their time and effort.

Focus groups were held in private spaces at the various organizations. All groups were facilitated by TF or EM and other co-authors and lasted between 40 and 60 min. Groups were audio-recorded and field notes were taken. In each group, participants viewed the opening sequences of SPARX and SPARX-R (in random order) and answered a schedule of open-ended questions (see **Table 2**). We used follow-up questions and reflective statements to explore and check understanding. Participants then completed a brief pen-and-paper questionnaire, which asked them 1) which program they thought they would prefer to use if they were feeling down, 2) which program they would prefer to use if they were not feeling down, and 3) whether they had ever felt down or low for more than a few days in a row. The questionnaire also requested brief demographic information and included an open space for further comments. Once data saturation was reached (i.e. no new information was discovered in data analysis) no further focus groups were held.

**Table 2:** Questions used to guide focus group discussion.

1. Do you think people your age get depression? Have you seen it? Do people your age know/recognize if they are depressed?
2. How do young people usually cope? What do they do to cope? Do they get help?
*First audio-visual excerpt shown (either SPARX or SPARX-R)*
3. What do you think about this program? What did you like? Not like?
4. Do you think it would be relevant for young people?
5. Do you think it would be relevant for young people who were feeling okay? What about for young people who were feeling down? What about for young people who had depression?
6. How would you feel about being asked to do this program?
*Second audio-visual excerpt shown (SPARX if shown SPARX-R previously or SPARX-R if shown SPARX previously)*
7. What do you think about this program?
8. Did you notice a difference? What was the difference?
9. Which version do you think is better to use with young people? What about if you were feeling good/a little down/had depression and/or were feeling very down? What are the pros/cons about each?
10. Any other comments?

### Participants

In total, 79 young people aged between 13 and 19 years old, from eight schools and one youth community organization (focus group five) participated in nine focus groups, as shown in **Table 3**. Forty-seven (59.5%) were female, the majority were between 15 and 17 years old (13 years, n = 3; 14 years, n = 8; 15 years, n = 12; 16 years, n = 20; 17 years, n = 23; 18 years or older, n = 10). The sample was ethnically diverse (11 Māori; 11 Samoan, Tongan or other Pacific Island; 8 Asian; 37 New Zealand European; 4 “other ethnicity”). Most (75.9%, n = 60) reported that they had suffered from feeling down or low for more than a few days in a row. The number of participants as well as proportions of males and females, and proportions of participants who reported feeling down or low for more than a few days in a row, varied among the focus groups as shown in **Table 3**.

**Table 3:** Participant demographics by focus group.

Group	*N*	Gender		Age						Ethnicity					Feeling low^a^	
		Male	Female	13	14	15	16	17	18+	Māori	Pacific	NZE^b^	Asian	Other	Y	N
1	10	4	6				1	7	2	2	1	6	1		9	1
2	6	1	3			2		2				4			4	0
3	4	1	3		1	1		1	1	2			1		1	1^c^
4	11	2	9				4	6	1		6		3	1	10	1
5	13	6	7				3	4	6	3	2	7		1	12	1
6	10	5	5	3	4	3						5		2	5	2
7	7	2	5			2	4	1		1	1	3	2		7	0
8	7	4	1^d^			3	2	1				5	1		4	2
9	11	3	8		3	1	6	1		3	1	7			8	3
**Total**	**79**	**28**	**47**	**3**	**8**	**12**	**20**	**23**	**10**	**11**	**11**	**37**	**8**	**4**	**60**	**11**

^a^Affirmative response to “have you ever suffered from feeling down or low for more than a few days in a row?”. ^b^New Zealand European. ^c^, 1 individual responded “not sure”, ^d^, 1 individual responded “prefer not to say”.

Row totals may not match due to missing data.

### Analysis

We used a general inductive approach (28) to analyze transcripts and open-response questionnaire comments. This approach is appropriate for interpreting content regarding relatively specific research or service delivery questions. First, TF, EM, and EH-W familiarized themselves with the data through repeated reading of the transcripts. They identified basic units of information and developed initial codes, which were clustered with other similar codes to create potential themes. Second, themes were refined by assessing contradictory points, subtopics and reviewing the essence of each theme. The researchers independently viewed the data, and drafted themes using the same process. Identified themes were then discussed among the coders and reviewed with co-authors. Differences were resolved by consensus and quotes encapsulating the themes were selected. Throughout the analysis process, the scripts were re-read to ensure that the findings remained true to the data.

Questionnaire responses were imported into IBM SPSS (Version 19). Simple descriptive statistics were generated but statistical testing was not carried out due the exploratory aims of the study and the small sample size. The Consolidated Criteria for Reporting Qualitative Research (COREQ) (29) guidelines were used to guide the reporting of the study.

## Results

We identified four themes relating to the relative acceptability and appeal of CCBT explicitly “for depression” and CCBT with more general wording (i.e., SPARX compared with SPARX-R): 1) naming depression is risky, 2) universality, 3) validation, and 4) choice. Quotes illustrating each theme are presented in **Table 4**. In addition to these four themes, an overarching theme “computerized therapy is accessible” was identified, which reflected a high level of interest from adolescents in computer programs as an approach for accessing help.

**Table 4:** Themes and example quotations.

Theme	Example quotes	Focus group
Computerized therapy is accessible	“You can just stay at home and relax with the computer.”	FG1
	“It’s good for [people] to not really have to leave their comfort zone to get help.”	FG8
	“It’s easier than [going to] talk to someone and sit down with them and go through all your problems with them.”	FG6
Naming depression is risky	“‘Depression’ is off-putting.”	FG4
	“I think [SPARX-R] is more on the safe side. You’re less likely to take offence to it.”	FG1
	“It kind of felt more belittling with (the guide) saying this game is to help people with depression. It made you feel worse about yourself for having this problem.”	FG8
	“[SPARX-R] doesn’t sound as severe … it’s kind of like everyone goes through this sort of thing. You know you’re not weird or having it labelled as [having] depression.”	FG5
Universality	“I think [SPARX-R] will reach out to more people.”	FG2
	“Even if they weren’t depressed and were just feeling sad, you could aim the game at them too and make them feel better as well as the people that are dealing with depression.”	FG4
	“It’s like, even if you are doing fine, SPARX-R can strengthen the skills. And I think that’s gonna appeal to everyone. Even if people are not completely depressed but just not feeling the best.”	FG5
	“I like that [SPARX-R] didn’t say ‘depressed people’, because not everyone comes to terms with the fact that they may be feeling depressed.”	FG4
Validation	“When you’re saying they’ve got hassles and stress and stuff, that’s kind of putting them down in [terms of] what they’re actually going through [if they are depressed].”	FG1
	“I know some people that are depressed and SPARX-R is like ‘oh, you’re stressed and stuff’. They take real offence to that because they know what they’ve got so they like people being straight up about it.”	FG1
	“I liked SPARX more because of the way it acknowledges you’re feeling depressed.”	FG8^a^
Choice	“At the end of the day it all depends on the person and how they carry their depression.”	FG4
	“You could make it that they answer questions before they get into it, that sort of tells you what space they’re in and whether … they already sort of feel like they have depression so then it’s okay to say it, or whether they’re just feeling a bit down.”	FG8

### Computerized Therapy Is Accessible

This theme reflected participants’ views that computerized therapy would be useful for adolescents as it is an easy way of getting help and does not require young people to speak with someone face-to-face. They considered the game-style interface of both versions (SPARX and SPARX-R) to be fun and much easier to access than other means of getting help for psychological issues. Speed and ability to get into “playing” quickly were noted as important. While participants considered both SPARX and SPARX-R to be somewhat “clunky” and “old school” they thought that they would still use it, as long as it was not too slow to play online.

### Naming Depression Is Risky

Participants considered the “depression language” used in SPARX to be “a little bit scary” and some thought that the explicit assumption that users “had depression” could make users feel worse. Many considered this approach more confronting than the “toned down” language used in SPARX-R, suggesting that the “abrupt” use of depression language may be off-putting, or even offensive, to youth who may not want to be labeled as “having depression” or are unsure whether they are depressed.

### Universality

Participants considered the language of SPARX-R more accessible than the “depression language” used in SPARX. They reported that the language used in SPARX-R was:

- Inviting for young people who were not depressed but were struggling with a range of issues (e.g., anger and stress);
- Inviting for those who might not realize they are depressed or, if they do realize, may want to keep this private; and
- Still relevant for those with depression.

Thus, participants reported that they would be more likely to recommend SPARX-R than SPARX to a friend, even if that friend had depression. Participants thought that “depression language” might “put [young people] off” getting the help they need. Counter to this, a minority of participants noted that, while SPARX-R was more accessible to a wider group, they enjoyed the direct approach and language used in SPARX, where the aim of helping people who might have depression is not hidden or couched in softer language. This ties in with the idea of validating young people’s experiences of depression.

### Validation

While it was generally agreed that SPARX-R was more appropriate for a wider audience, a minority of participants considered that the language in SPARX-R could be interpreted by some who had experienced clinical depression as belittling their experience. They thought that the direct language used in SPARX may confirm that this program was appropriate for them. However, provided with a scenario where only limited resources were available, participants considered SPARX-R to be a better option than SPARX.

### Choice

Participants expressed the perspective that both approaches (i.e., depression-specific CCBT and a more general CCBT program) have positive and negative attributes. There was general agreement that both had value and users should be offered different versions depending on their preferences, the severity of their symptoms, and the route *via* which they had been offered the program (e.g., internet search *versus* recommendation by a clinician).

In questionnaire responses, the majority of participants preferred SPARX-R to SPARX, whether or not they had a history of feeling low for more than a few days in a row (see **Table 5**). The majority considered that if they were feeling down or depressed, they would still prefer SPARX-R or would like both versions equally.

**Table 5:** Questionnaire responses.

	Which program did you like more/prefer?		
	SPARX	Both equally/don’t mind	SPARX-R
Have you ever suffered from feeling low for more than a few days in a row?			
Yes	11 (19%)	8 (14%)	40 (68%)
Not Sure	0	0	1
No	2	2	7
Total^a^	13 (18%)	10 (14%)	48 (68%)
If I was feeling down or depressed I think I would prefer^b^…	25 (33%)	12 (16%)	38 (51%)
If I was NOT feeling down or depressed I think I would prefer^c^…	14 (19%)	14 (19%)	47 (63%)

^a^Missing data n = 8; ^b^Missing data n = 4; ^c^Missing data n = 4.

## Discussion

In this exploratory study we found that adolescents considered the way a CCBT program is presented and the language used is important for its appeal. While both SPARX computerized therapy “for depression” and SPARX-R computerized therapy “for young people generally” were received favorably, participants considered that the less clinical wording of SPARX-R would appeal to a broader range of adolescents. They suggested that this approach would hold greater appeal for those who 1) did not have depression, 2) did not recognize themselves as depressed, and 3) were uncomfortable identifying as depressed, while retaining relevance for those who did identify as depressed. Many participants expressed a preference for user choice between both options, as naming depression explicitly could be validating for some. However, should only one option be offered, they preferred the more broadly focused approach. Although these findings are from just one study and one comparison, they suggest that developers should give careful consideration to how CCBT for young people is presented.

Computerized therapies hold promise for reducing the large treatment gap for depression, but analyses suggest that such tools are yet to achieve their potential impact (8, 30–32). Systematic analyses highlight that clinical support can improve retention in CCBT (32). However, over half of young people with clinically significant symptoms do not seek professional help and clinically supported approaches will not address this. Gulliver, Griffiths, and Christensen (33) reviewed qualitative and quantitative studies, and found that adolescents identified the most significant barriers to help-seeking as: perceived stigma and embarrassment, difficulty recognizing symptoms (i.e., poor mental health literacy), and a preference for self-reliance. We have previously identified that even looking up a website “for depression” in private can be off putting for some adolescents (34). Computerized approaches that avoid the terminology linked to diagnostic categories may help to reduce the barriers associated with stigma and embarrassment, whether a young person is seeking help *via* a professional or on their own (e.g. *via* an internet search). It is noteworthy that many popular contemporary programs and apps focus on personal self-help or development and do so without naming specific psychiatric disorders. They instead highlight specific challenges (such as sleep), or aspirations, such as improved mood (35).

Universal or selective interventions have real potential in the face of limited help seeking amongst adolescents. To date, school- and education-setting-focused depression prevention interventions have shown promising results (20, 21). Ideally, users might choose a program (or, in computer science terms, a “skin” or pre-set appearance package) that appeals to them. As this option is not yet commonly available, interventions should be framed in a way that is welcoming and relevant for all, without minimizing disorder or causing other harms. In relation to prevention trials, it is useful to compare the two large high school-based studies of SPARX. One was a cluster RCT comparing SPARX-R with an online control program (26), and the other compared a Dutch translation of SPARX with a routine monitoring control condition and group-based CBT (27). The cluster RCT identified positive effects for SPARX-R for the prevention of depressive symptoms (26), while the Dutch trial did not find a greater reduction in subclinical symptoms in the SPARX condition than the other two conditions (27). There are a number of differences between these two trials that could account for their contrasting results. For instance, SPARX-R in Australia had a sample size of 540 students (63.1% female, mean age = 16.7 years) whereas the Dutch trial had a sample of 208 students (100% female, mean age = 13.4 years). Given the differences in prevalence of depression by age (36), the study in the Netherlands may have been underpowered. Differences in presentation between the two versions of SPARX may also have had an impact. For example, the professional voice actor who voiced the Guide in both SPARX and SPARX-R was carefully selected because of his warm empathic voice and young people have previously commented that they felt the guide cared for them (17). Different voice actors were used in the Dutch language version of SPARX. It is also possible that the contrasting findings regarding effectiveness could be due in part to the differences in language used to frame SPARX and SPARX-R, and resulting differences in user appeal. This hypothesis could be explored in further research. It would be valuable to directly contrast clinically and less clinically focused versions of programs in future studies.

### Strengths and Limitations

We sampled a small, unique population group (young people living in urban parts of Auckland, New Zealand) and examined versions of one particular CCBT program (SPARX/SPARX-R). Other groups and other programs might yield different findings. However, our sample was diverse in terms of age, gender, and ethnicity. Co-designers of SPARX (TF, KS, and MS) carried out some of the focus groups, which may have led to a social desirability bias. To constrain this, honest and frank discussion was actively encouraged and participants completed anonymous questionnaires at the end of focus groups. Importantly, the sample was from a non-clinical population and depressive symptomatology was not assessed using a validated measure, although a large proportion of the participants reported having experienced periods of significant low mood. This is an important consideration, as preference between SPARX and SPARX-R can be expected to be influenced by experience with depression. Further research using a clinical sample or a validated depression measure would allow for comparisons of uptake, as well as adherence and effectiveness, between clinical (and potentially help-seeking) young people and youth from the general population.

Our focus groups varied in size from 4 to 13 persons and included differing proportions of males, females, and participants who had felt down or depressed. Guidance for focus group size often ranges from 4 to 12 participants (37), or more narrowly, for example from 6 to 10 participants (38). Our written feedback sheet did allow an opportunity for additional individual comments that might not have been made in groups; however, more standard sized groups might have allowed richer discussion.

## Conclusions

This exploratory study suggests that the language used to frame the purpose of CCBT has implications for its appeal to adolescents. Some young people may perceive the term “depression” negatively, as well as stigmatizing and exclusionary to those struggling with less severe issues, whereas others may perceive it positively, as validating of a young person’s depressive experiences. Offering different terminology to meet diverse personal preferences is ideal, but where this is not possible and the therapy is designed for widespread use amongst youth more generally, our results suggest that adopting less clinically orientated or diagnostically focused language may broaden appeal.

## Data Availability Statement

The datasets for this study will not be made publicly available because data include statements made by individual young people and the ethics approval obtained by the University of Auckland Human Subjects Ethics Committee does not allow this to be shared.

## Ethics Statement

This study was carried out in accordance with the recommendations of the University of Auckland Human Participants Ethics Committee with written informed consent from all subjects. All subjects gave written informed consent in accordance with the Declaration of Helsinki. The protocol was approved by the University of Auckland Human Participants Ethics Committee (Reference 2015/014991).

## Author Contributions

SM, KS, MS, ML, and TF developed and tested SPARX. TF identified the possible need for SPARX-R and developed this with SM and KS and others. TF and EM planned the focus group study. TF and EM led the focus groups with input from EH-W, KS, and MS. TF, EM, and EH-W carried out the initial analyses of results and then refined these with all co-authors. TF, EM, EH-W, and LB drafted the paper. All authors contributed to the paper and confirmed the final content.

## Conflict of Interest

SM, KS, TF, MS, and ML are co-developers of SPARX. The intellectual property for SPARX is held by Uniservices at the University of Auckland and the co-developers could benefit financially from licensing or profit generated from SPARX outside of New Zealand.

The remaining authors declare that the research was conducted in the absence of any commercial or financial relationships that could be construed as a potential conflict of interest.
